# Dr. Nott’s Munificence

**Published:** 1854-01

**Authors:** 


### MUNIFICENCE OF DR. NOTT.

The following statement from the *Albany Evening Journal*, in reference to a great and good man, is here given at length, as bearing directly on one of the main objects of this Journal, THE PRESERVATION OF THE HEALTH OF STUDENTS. It is a step forward in the right direction, for which the Editor has spoken and written for nearly twenty years, to wit, the establishment of a professorship in colleges, for the express purpose of imparting instruction in reference to the preservation of the constitutions of the young. It carries with it more than ordinary weight, since the importance of it has forced itself on the mind of the President of a college, from the observations of half a century. It is within a day or two that I read a notice of the death of a young gentleman, and with a change of name only it would be an appropriate obituary of thousands of others. "He was the son of the pastor of the old church, who had educated his children, and they were noble men. He was a great favorite, and deservedly so. He was not well when he returned from college after graduating, and after a few weeks of struggling, he gave up entirely, and *lay down in his father's house to die*. It was a terrible blow to the father and the family." And well it was. How terrible! none but a father can ever know. And it is to prevent the re-occurrence of such incidents, by hundreds every year, that this publication is undertaken, and that Dr. Nott has founded a perpetual professorship in the last item but one of his princely donation:

"Albany, Dec. 29, 1853.

"We spoke yesterday of a day of full and complete vindication for Dr. Nott. It has come.

"There was a time, not long since, when it seemed possible that vague charges, maliciously originated and ignorantly spread and prosecuted, might be allowed to outweigh, in the public estimation, the acts of a long, upright and useful life. That time, we are glad to be convinced, is not only passed, but, in the facts given below, there is a guarantee against it ever returning.

"It is just fifty years since Dr. Nott was called from the Presbyterian Church in Albany to the Presidency of Union College, then a feeble and puny institution, struggling for its very existence. He gave up the brilliant professional career that was opening before him, and devoted his time and his great abilities to the College, and through it to the advancement of Christian Education. How well he has succeeded is best testified by the thousands who have profited by his instructions. There is hardly a school district of the State, or a Church, or a Court of Justice, or a Legislative Session, that has not at one time or another felt his teachings through the divines, the lawyers, the teachers and the statesmen, that have been his pupils. His eulogy is not written, but living and breathing around him. And, in the meantime, the College has gradually become the largest, the richest, and the most celebrated, west of the New England line, and not inferior to those two which it has taken New England centuries to build.

"At the same time he made himself known as an inventor and author, and by laborious research and industry has been amassing a large private fortune. But this, also, he has jealously kept sacred, not for himself, but for the cherished objects of his life. Grown to almost princely proportions, he uses it now, in accordance with his long-entertained purpose, in a series of endowments that will place Union College above every similar institution in the land.

"The action of the Board of Trustees, at their meeting at Schenectady yesterday, explains how this is to be done, and how it is received. At some future time we hope to give the report in a more extended and complete form. The invitation in the concluding resolution will be responded to by the assembling of an army of graduates for congratulation, not only of their venerable Preceptor, but of the College, the State, and themselves.

"'This Board having witnessed, for several years past, the unceasing efforts made to impair the public confidence in this institution, and to injure the character and destroy the usefulness of our distinguished President, have, with him, waited their time with full confidence in an ultimate and triumphant result. This day, in pursuance of a determination formed and expressed more than twenty years ago, and the effectual accomplishment of which was many years since secured by the proper legal papers to take effect in the event of his unexpected decease, Dr. Nott has delivered to this Board, in trust, for the use of the College, money, securities and property of the estimated value of more than six hundred thousand dollars. This result, the fruit of individual skill and far-sighted policy, with donations previously made, show the noble disinterestedness which has marked his whole administration of the affairs of Union College, and which entitles him to the highest credit and honor, and to the lasting gratitude of all friends of education, and of the amelioration of our race; therefore,

"'*Resolved*, That the Trustees representing the College, and as individuals feeling a deep interest in the cause of education, tender to our venerable President our warmest thanks for his noble and disinterested conduct, for the moral courage and firmness with which he has met the assaults made upon his character, and for his munificent endowment of the institution committed to our charge.

"'*Resolved,* That we earnestly request all the graduates of Union College to meet us at the next annual commencement, and unite in congratulations to Dr. Nott at the then close of fifty years since he entered on his duties as President, and to rejoice with him and with us in the prosperity of this institution, to the advancement of which he has so successfully devoted the energies of a great mind for the thus unexampled period of half a century.'

"The following are the endowments. The several sums are to form a perpetual fund, the income only being used for the various purposes:

| | |
|---|---|
| For the establishment of nine Professorships, $1,500 each, per annum, | $225,600 |
| Six Assistant Professorships or Tutorships, at $600 per annum, | 60,000 |
| Observatory, | 20,000 |
| Sixty-eight Auxiliary Scholarships, | 50,000 |
| Fifty Prize Scholarships for under graduates, | 50,000 |
| Nine Prize Fellowships for graduates, $300 each, per annum, | 45,000 |
| Cemetery and Pleasure Grounds, | 20,000 |
| Philosophical, Mathematical and Chemical Apparatus, | 10,000 |
| Text Books, | 5,000 |
| Scientific, Classical, Philosophical, Theological, Medical and Law Books, | 30,000 |
| Cabinet of Geological Specimens, | 5,000 |
| Historical Medals, Coins, Maps, Paintings, and other Historical Memorials, | 5,000 |
| Lectures on the Dangers and Duties of Youth, especially Students; the Development and Preservation of the Physical, Intellectual and Moral Constitution of Man; Preservation of Health, and on the Laws of Life, | 10,000 |
| To meet taxes, liens, assessments, incumbrances, insurance, and compensation to Visitors, and to make up any deficiencies in the income of any of preceding principal sums, so as to secure the attainment of the objects and purposes designed, | 75,000 |
| Total, | $610,000 |

"There are to be five Visitors appointed, charged with the duty of acting in connection with the Trustees, and seeing that these trusts are faithfully carried out."

